# Pseudoathetotic Pseudodystonia as a Manifestation of Isolated Medullary Demyelination in Neuromyelitis Optica Spectrum Disorder

**DOI:** 10.5334/tohm.1153

**Published:** 2026-02-10

**Authors:** Prachi Mohapatra, Lekshmi Sambhu Hema, Aditya Mahadevan, Divyani Garg, Ayush Agarwal, Awadh Kishor Pandit, Ajay Garg, Achal Kumar Srivastava, Divya M. Radhakrishnan

**Affiliations:** 1 Department of Neurology, All India Institute of Medical Sciences, New Delhi, India; 2 Department of Neuroimaging and Interventional Neuroradiology, All India Institute of Medical Sciences, New Delhi, India

**Keywords:** Pseudoathetosis, Pseudodystonia, Medullary demyelination, Movement disorder mimic, Neuromyelitis optica spectrum disorder

## Abstract

**Background:**

Pseudoathetosis and pseudodystonia are rare sensory-driven hyperkinetic movement disorders that may mimic primary dystonia. Although these manifestations have been reported in neuromyelitis optica spectrum disorder (NMOSD), they are typically associated with cervical spinal cord lesions, and occurrence due to isolated medullary demyelination is extremely uncommon.

**Case Report:**

A 32-year-old woman with aquaporin-4-positive NMOSD developed pseudoathetotic movements with dystonic-appearing posturing of both hands following an isolated demyelinating lesion of the medulla with normal spinal cord imaging. The abnormal movements persisted after corticosteroids but resolved completely following plasmapheresis and rituximab.

**Discussion:**

This case expands the known anatomic spectrum of NMOSD-associated movement disorders by demonstrating isolated medullary demyelination as a rare substrate for pseudoathetotic pseudodystonia and emphasizes the importance of recognizing sensory-driven hyperkinetic movements to ensure timely immunotherapy.

## Introduction

Pseudoathetosis and pseudodystonia are related sensory-motor integration disorders that can mimic primary movement disorders. Pseudoathetosis is characterized by slow, writhing movements of the distal extremities caused by impaired proprioceptive input, most noticeable when visual compensation is removed (e.g., when the eyes are closed). In contrast, pseudodystonia refers to abnormal postures or patterned movements that resemble dystonia but originate from non-basal ganglia mechanisms [1]. Although pseudoathetosis is typically associated with dysfunction of proprioceptive pathways in the spinal cord or peripheral nerves, and pseudodystonia with diverse structural or systemic etiologies, their coexistence suggests disruption of sensory-motor integration. These phenomena have been reported in cervical spinal cord lesions but are rarely observed with isolated medullary pathology with normal spinal cord imaging. We report a patient with aquaporin-4 positive neuromyelitis optica spectrum disorder (NMOSD) presenting with combined pseudoathetosis and pseudodystonia caused by isolated medullary demyelination, illustrating how brainstem involvement can masquerade as a primary movement disorder and emphasizing the importance of recognizing this reversible, immune-responsive presentation.

## Case Report

A previously healthy 32-year-old woman initially presented with persistent hiccups, nausea, and intermittent vomiting lasting six weeks. She was treated symptomatically with oral pantoprazole and ondansetron. Subsequently, her condition worsened, with progressive weakness beginning in the distal parts of all four limbs and gradually moving proximally. She also reported paraesthesias in her limbs, increasing unsteadiness while walking, and difficulty with fine motor tasks. Two weeks later, she developed continuous, slow, writhing movements and abnormal posturing of both hands and wrists, predominantly involving the fingers. The movements interfered with feeding and writing and persisted even at rest. These bilateral involuntary movements demonstrated mixed phenomenology, with pseudoathetosis characterized by slow, undulating movements that worsened with eye closure, reflecting impaired proprioceptive feedback, and dystonic-appearing posturing consistent with “pseudodystonia” (Video 1). The movements were not voluntarily suppressible, lacked a sensory trick, and were exacerbated by tactile stimulation or anxiety. She had no history of exposure to dopamine receptor-blocking or antidopaminergic medications during the course of her illness.

**Video 1:** Pre- and Post-Treatment Video of the Patient. Phenomenology 0:00–0:08 – Bilateral slow, writhing finger and hand movements at rest. 0:08–0:16 – Marked increase in amplitude and dystonic posturing with eyes closed. 0:17–0:26 – Post-plasmapheresis: complete resolution of movements. 0:26–0:29 – No re-emergence on eye closure.

Neurological examination revealed mild to moderate quadriparesis (MRC 4/5 proximally, 3/5 distally) with greater distal involvement, impaired vibration and joint-position sense up to the elbows and hips, and brisk deep tendon reflexes with mute plantar responses. No sensory level was demonstrable. Coordination testing showed bilateral dysmetria and impaired finger-nose and heel-knee-shin tests. Cranial nerves and higher mental functions were normal.

**Figure 1:**
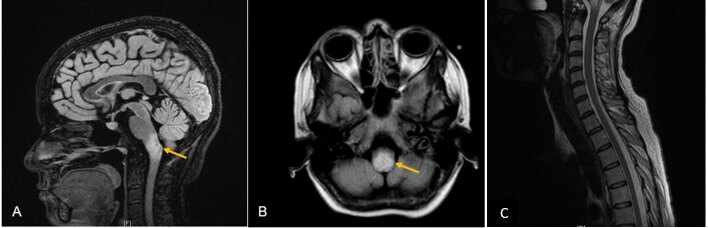
**MRI demonstrating isolated medullary involvement. (A)** Sagittal T2-FLAIR image showing a focal hyperintense lesion in the dorsal medulla (arrow), with no signal abnormality in the cervicomedullary junction. **(B)** Axial T2-FLAIR image demonstrating the corresponding medullary hyperintensity (arrow). **(C)** Sagittal T2-weighted image of the cervico-dorsal spinal cord showing no signal abnormality.

Routine hematologic and biochemical investigations, autoimmune and infectious serologies, and vitamin profiles were within normal limits. [Vitamin B12: 914 pg/mL (reference range 197–771), serum folate: 8.7 ng/mL (reference range 3.1–17.5)]. All laboratory investigations are summarized in Table 1. MRI of the brain revealed T2/FLAIR-hyperintense lesions in the dorsal medulla extending to the area postrema, with patchy gadolinium enhancement (Figure 1A, 1B). No supratentorial lesions were seen. MRI of the spinal cord was unremarkable (Figure 1C). Serum aquaporin-4 antibodies were strongly positive, confirming a diagnosis of neuromyelitis optica spectrum disorder (NMOSD) presenting with an acute brainstem syndrome.

**Table 1:** Baseline laboratory investigations of the patient.


INVESTIGATIONS	PATIENT’S VALUES	REFERENCE RANGE

**Hemoglobin (g/dl)**	12.9	12–15

**Total Leukocyte count (× 10^3/µL)**,	9.8	4–10

**Neutrophil %/Leukocyte %**	62/25	40–80/20–40

**Platelet count (× 10^3/µL)**	273	150–410

**Prothrombin time(s)/INR**	13.5/1.1	11–16/0.8–1.1

**ALT/AST (U/L/ U/L)**	27/48	14–36/10–49

**Creatinine/Urea (mg/dl/mg/dl)**	0.6/37	0.52–1.04/15–42

**HbA1C(%)**	5.2	<5.7

**Lipid profile/T3/T4/TSH**	Normal	

**Vitamin B12 (pg/ml)**	914	197–771

**Serum Folate (ng/ml)**	8.7	3.1–17.5

**Serum Homocysteine (umol/L)**	14.7	0–15

**ANA/ENA/ANCA (IIF)**	Negative	

**Serum ACE levels**	Normal	

**HIV/HbsAg/anti-HCV**	Negative	

**Serum Anti-Aquaporin 4 IgG (Fixed CBA-IIF)**	**Strong positive**	

**Serum MOG IgG (Fixed CBA-IIF)**	Negative	

**CSF- cells/uL, Neutrophil %/Lymphocyte %**	Nil	0-5 cells/uL

**CSF- protein (mg/dl)**	36	15–45

**CSF-sugar (mg/dl)**	64/110	40–70

**CSF- gram stain, culture, India Ink, Cryptococcal Antigen, VDRL, Acid fast bacilli staining, Gene Xpert, Malignant cytology**	Negative	

**Serum Paraneoplastic Panel (Immunoblot)** **Anti Hu, Anti Ri, Anti Yo, Anti CV2, Anti Ma2/Ta, Anti Amphiphysin, Anti GAD 65, Anti Zic 4, Anti titin, Anti Recoverin**	Negative	

**Serum and CSF Autoimmune Panel (Fixed CBA-IIF)** **NMDA, AMPA 1,2, CASPR2, LGI1, GABA B**	Negative	


*CBA-IIF: Cell-Based Assay- Indirect Immunofluorescence, ANA: Anti-Nuclear Antigen, ENA: Extractable Nuclear Antigen, ANCA: Anti-Neutrophilic Cytoplasmic Antibody, CSF: Cerebrospinal Fluid, MOG- Myelin Oligodendrocyte Glycoprotein, NMDA: N- Methyl-D-Aspartate, AMPA: α-amino-3-hydroxy-5-methyl-4-isoxazoleproprinoic acid, CASPR2: Contactin associated protein type 2, LGI1: Leucine Rich Glioma Inactivated Protein-1, GABA-B: Gamma-Aminobutyric Acid-B.

She received intravenous methylprednisolone 1 g/day for 5 days, resulting in partial improvement in limb strength and resolution of nausea and hiccups, though pseudoathetotic movements persisted. Subsequently, five cycles of plasmapheresis led to complete resolution of abnormal movements and marked sensory recovery (Video 1). Rituximab 1 g, administered as two doses two weeks apart, was used for induction therapy. At 6-month follow-up, she remained relapse-free with sustained remission of pseudoathetosis and pseudodystonia and no new MRI lesions.

## Discussion

The co-occurrence of pseudoathetosis and pseudodystonia highlights disruption of sensory-motor integration rather than a primary dystonia phenotype [1]. While movement abnormalities frequently occur in NMOSD, they mostly present as tonic spasms. Sensory-mediated, writhing movements such as pseudoathetosis are rarely seen [2]. Pseudoathetotic movements are typically associated with posterior spinal cord involvement or large-fibre peripheral neuropathy. Isolated medullary or medulla-predominant involvement presenting as pseudoathetosis is uncommon, with only a single case reported so far describing pseudoathetosis after a right medullo-pontine hemorrhage, characterized by proprioceptive loss and contralateral pseudoathetotic hand movements [3]. Additionally, ponto-mesencephalic lesions have been reported to produce a spectrum of abnormal movements, including hemidystonia, choreodystonia, athetoid movements with tremor, and dystonia associated with impaired proprioception, highlighting the ability of focal brainstem lesions to generate pseudodystonic and sensory-driven hyperkinetic phenomenology [4,5,6,7].

Within NMOSD, pseudoathetosis has been documented in only two previous reports: one involving cervical myelitis and aquaporin-4 positivity, showing posterior column lesions from C1–C8 that resolved with steroids [8], and another presenting as a complex hyperkinetic movement disorder with pseudoathetosis secondary to a posterior cervical cord lesion (C3–C6), which improved with plasmapheresis and rituximab [9]. Our case differs by demonstrating isolated medullary demyelination with normal cord imaging, thus expanding the known neuroanatomical substrate of pseudoathetosis in NMOSD. The medulla contains several structures critical to proprioceptive relay and motor modulation. Demyelinating injury involving the gracile and cuneate nuclei, medial lemniscus, or inferior cerebellar peduncle can distort sensory input to thalamic and cortical circuits, leading to abnormal motor output characterized by slow, irregular movements. Concurrent disruption of the dentato-rubro-olivary and pallidal-pedunculopontine pathways may further produce dystonic-appearing posturing, or pseudodystonia, accounting for the hybrid sensory-extrapyramidal phenomenology observed in our patient [4].

Our case highlights that pseudoathetosis and pseudodystonia in demyelinating diseases reflect active inflammatory injury rather than permanent basal ganglia dysfunction. Therefore, immunotherapies such as corticosteroids, plasmapheresis, or B-cell depletion can lead to complete recovery if started early, while symptomatic dystonia treatments (anticholinergics, botulinum toxin) are ineffective. Recognizing objective sensory impairment and identifying a structural lesion affecting sensory pathways are central to distinguishing pseudoathetosis and pseudodystonia from primary movement disorders and for initiating prompt immunotherapy. While abnormal movements may become more apparent or worsen with eye closure due to loss of visual compensation, this feature alone is not specific and can also be observed in primary dystonia. Rather, dystonic-appearing posturing that lacks a sensory trick and occurs in the setting of demonstrable sensory pathway involvement should favor a diagnosis of pseudodystonia over true dystonia.

## Conclusion

In summary, this case expands the clinical and anatomical spectrum of NMOSD by demonstrating that isolated medullary demyelination can manifest as pseudoathetotic pseudodystonia. Recognizing this unique, immune-responsive phenotype is essential to prevent misdiagnosis as a primary movement disorder and to start timely immunotherapy for complete recovery.

## References

[1] Berlot R, Bhatia KP, Kojović M. Pseudodystonia: A new perspective on an old phenomenon. Parkinsonism Relat Disord. 2019;62:44–50. DOI: 10.1016/j.parkreldis.2019.02.008 30819557

[2] Bringel LAF, Lima PLGSB, Rodrigues PVF, et al. Movement Disorders in Neuromyelitis Optica Spectrum Disorder: A Systematic Review. Mov Disord Clin Pract. Published online September 2, 2025. DOI: 10.1002/mdc3.70339 PMC13042436 40891169

[3] Torres L, Cosentino C, Suárez R. Pseudoatetosis por hemorragia bulboprotuberancial [Pseudoathetosis after medullar and pontine hemorrhage]. Rev Neurol. 2002;34(1):89–90. DOI: 10.33588/rn.3401.2001219 11988895

[4] Park CW, Chung SJ, Sohn YH, Lee PH. A Case of Abnormal Postures in the Left Extremities after Pontine Hemorrhage: Dystonia or Pseudodystonia? J Mov Disord. 2020;13(1):62–65. DOI: 10.14802/jmd.19074 31986870 PMC6987531

[5] Tan EK, Chan LL, Auchus AP. Hemidystonia precipitated by acute pontine infarct. J Neurol Sci. 2005;234(1-2):109–111. DOI: 10.1016/j.jns.2005.03.046 15935386

[6] Kim HJ, Cho YJ, Cho JY, Hong KS, Jeon BS. Choreodystonia in a patient with hypertrophic olivary degeneration after pontine tegmental hemorrhage. Mov Disord. 2008;23(6):920–922. DOI: 10.1002/mds.21959 18311825

[7] Loher TJ, Krauss JK. Dystonia associated with pontomesencephalic lesions. Mov Disord. 2009;24(2):157–167. DOI: 10.1002/mds.22196 18951533

[8] Seok HY, Jang SH, You S. Neuromyelitis optica spectrum disorder presenting with pseudoathetosis. J Clin Neurol. 2018;14(1):123–125. DOI: 10.3988/jcn.2018.14.1.123 29629549 PMC5765249

[9] Aguiar RF, Nóbrega PR, Veras SRO, et al. A complex hyperkinetic movement disorder responsive to immunotherapy in a patient with neuromyelitis optica. Mov Disord Clin Pract. 2020;7(7):695–698. DOI: 10.1002/mdc3.13034 32775518 PMC7396851

